# Expression of Yin Yang 1 in cervical cancer and its correlation with E-cadherin expression and HPV16 E6

**DOI:** 10.1371/journal.pone.0193340

**Published:** 2018-02-22

**Authors:** Wanxue Wang, Zhenni Yue, Zhengping Tian, Yiran Xie, Jiamiao Zhang, Yuanping She, Bing Yang, Yuan Ye, Yihua Yang

**Affiliations:** 1 Center of Reproductive Medicine, the first Affiliated Hospital of Guangxi Medical University, Nanning, China; 2 Center of Reproductive Medicine, the Affiliated Hospital of Guilin Medical College, Guilin, China; 3 Department of Obstetrics and Gynecology, the Affiliated Hospital of Guilin Medical College, Guilin, China; Georgetown University, UNITED STATES

## Abstract

The molecular mechanisms of normal cervical squamous epithelium advancing to cervical intraepithelial neoplasia (CIN) and eventually to cervical squamous cell carcinoma (CSCC) are largely unknown. This study explored abnormal expression of Yin Yang 1 (YY1) in cervical cancer and its correlation with the expression of E-cadherin and human papillomavirus (HPV) 16 E6. YY1, E-cadherin and HPV16 E6 expression were detected by immunohistochemistry in 90 cervical tissue specimens collected from 30 patients with hysteromyoma, 15 patients with CIN I, 15 patients with CIN II-III, and 30 patients with CSCC. The H-score method was employed to measure the expression of YY1, E-cadherin and HPV16 E6. Increased expression of YY1 and HPV16 E6, and the decreased expression levels of E-cadherin were strongly associated with malignant transformation of the cervical epithelium and the histological progression of CSCC. The expression of YY1 in cervical tissues was inversely correlated with E-cadherin expression, and positively correlated with HPV16 E6 expression. Expression of YY1 in CSCC tissues was not significantly correlated with tumor differentiation, but was significantly correlated with an advanced clinical stage of CSCC. These results suggest that up-regulation of YY1 is closely associated with the progression of CSCC, and YY1 may play an important role in the pathogenesis of cervical cancer by modulating the expression of E-cadherin and HPV16 E6.

## Introduction

Cervical cancer is a common malignancy in women, of which the most frequent pathological type is cervical squamous cell squamous carcinoma (CSCC). Current research suggests that cervical cancer pathogenesis is associated with the persistent infection of high-risk human papillomavirus (HPV) [1, 2]. However, the pathogenesis of cervical cancer is a complex process, which involves multiple genes and gene-factors, thus, high-risk persistent HPV infection is not the only factor in cervical cancer progression and pathogenesis. Apart from high-risk HPV infection, studies have demonstrated that various tumor-suppressors, oncogenes and tumor growth factors are involved in these disease processes [3, 4]. In China, the incidence and mortality of cervical cancer remains high, and the age of cervical cancer onset tends to be younger [5]. In this regard, cervical cancer has become a serious threat to female health. Investigation of cellular factors involved in the pathogenesis and progression of cervical cancer may provide novel strategies for management of this malignancy.

Yin Yang 1 (YY1) is a ubiquitously distributed transcriptional factor belonging to the GLI-Kruppel class of zinc finger proteins. YY1 can act as either transcriptional activators or repressors in gene expression regulation [6, 7]. YY1 was found to be oncogenic in various types of cancers, such as breast cancer, prostate cancer and lymphomas [8, 9]. Specifically, studies have shown that YY1 is up-regulated in cervical cancer [10] in addition to negatively regulating the expression of E-cadherin in the pathogenesis of breast cancer [11]. E-cadherin acts as a tumor-suppressor and is critical for the formation and maintenance of adherent junctions in areas of epithelial cell-cell contact. Most tumors have abnormal cellular architecture, and loss of tissue integrity leads to local invasion. Thus, loss of E-cadherin critical cellular function correlates with increased invasiveness and metastasis of tumors [12, 13]. Recent studies have demonstrated that low E-cadherin expression is a negative, independent prognostic factor in patients with cervical cancer [14].

The HPV16 E6 gene has been shown to be one of major oncogenes in high-risk persistent HPV infection and closely associated cervical cancer pathogenesis. HPV16 E6 serves as an important biomarker for the development of cervical cancer. Mechanistically, HPV16 E6 was found to promote the pathogenesis and progression of cervical cancer via complex mechanisms, such as suppressing p53 expression, regulating Daxx expression, and enhancing telomerase activity [15–17]. Studies have demonstrated that YY1 negatively regulates the HPV16 E6 expression in cervical cancer [18], suggesting that YY1 may target many downstream effectors to regulate the pathogenesis and progression of cervical cancer.

Effects of YY1 on the expression of E-cadherin and HPV16 E6 has been documented in previous studies [11, 18]. Previously, we also demonstrated the novel miR-193a-5p-YY1-APC regulatory axis in regulation of endometrioid endometrial adenocarcinoma progression [19], however, to the best of our knowledge, the expression of YY1, E-cadherin and HPV16 E6, as well as the correlations between them, have not been reported on normal cervical tissues, cervical intraepithelial neoplasia (CIN) tissues and CSCC tissues. In this study, the expression of YY1, E-cadherin and HPV16 E6 in normal cervical tissues, CIN tissues and CSCC tissues was determined by immunohistochemistry (IHC). The correlations between YY1 and E-cadherin as well as HPV16 E6 were also analyzed. Our findings may provide new insights into understanding YY1-mediated pathogenesis of cervical cancer.

## Materials and methods

### Patients and clinical samples

A total of 90 patients, 30 patients with hysteromyoma, 15 patients with CIN I, 15 patients with CIN II-III, and 30 patients with CSCC were enrolled in this study. CIN or CSCC tissues were collected from patients with CIN or CSCC receiving cervical biopsy and hysterectomy, and normal cervical tissues were collected from patients with hysteromyoma receiving hysteromyoma resection, and all the patients were HPV 16 DNA positive. The clinical characteristics of patients are described in the results section. All the clinical samples were collected between May 2013 and April 2015 at the Affiliated Hospital of Guilin Medical University. All clinical specimens were kept in tissue paraffin blocks. Each pathological diagnosis was reviewed in a double-blind manner by two experienced pathologists. The clinical stage of cervical cancer was defined according to criteria from the International Federation of Gynecology and Obstetrics in 2009 [20]: 12 cases were diagnosed as Stage I_A_-I_B1_, and 18 cases were diagnosed as Stage I_B2_-II_A_. With regards to tumor differentiation, 13 cases were well or moderately differentiated, and 17 cases were poorly differentiated. Patients who were pregnant, or who had received a previous cervical therapy or preoperative treatment, or who were diagnosed other types of malignancies were excluded for the study. This study was reviewed and approved by the Ethics Committee of the First Affiliated Hospital of Guilin Medical University, and written informed consent was obtained from all patients.

### Immunohistochemistry

Sections measuring 4-μm were sectioned from the paraffin-embedded cervical tissue sample blocks. The tissue sections were de-paraffinized in xylene for 10 min, fixed using 100% ethanol for 5 min, and then dehydrated with 95%, 85% and 75% ethanol. Antigen retrieval was performed by placing the tissue sections in sodium citrate buffer (for YY1 and E-cadherin detection) or ETDA buffer (for HPV16 E6 detection). Application of a high voltage for 3 min was then followed by natural cooling. Endogenous peroxide activity was reduced by placing the tissue sections in the 3% hydrogen peroxide in methanol for 10 min at room temperature. The sections were then washed twice with phosphate buffered saline (PBS) and incubated with the primary antibodies: mouse anti-YY1 monoclonal antibodies (1:100, Santa Cruz, Dallas, USA), rabbit anti-E-cadherin polyclonal antibodies (1:100, Santa Cruz), rabbit anti-HPV16 E6 polyclonal antibodies (1:100, Santa Cruz) overnight at 4°C. The sections were then washed with PBS and incubated with corresponding secondary antibody for 30 min at room temperature. The sections were then rinsed with PBS and incubated with diaminobenzidine (DAB) chromogen for 3–5 min. Finally, the slides were counterstained with 10% hematoxylin, and stained images were captured using a microscope with a digital camera.

### Immunoreactive score (H-score)

The specimen signal strength was scored manually by two experienced pathologists using histology slide images under 400x microscope and the following scale: 0, no signal above background; 1, weak signal; 2, moderate signal; and 3, strong to very strong signal. H-score was calculated using the following formula: H-score = (% cells not stained × 0) + (% cells stained weak × 1) + (% cells stained moderate × 2) + (% cells stained strong × 3).

### Statistical analysis

All the statistical analyses were performed by using the statistical software, SPSS version 18.0 (Chicago, USA). Significant difference between two groups were analyzed by t-test, and significant difference among more than two groups was analyzed by one-Way ANOVA. The correlation analysis was performed using the Spearman rank correlation test. P<0.05 was considered statistically significant.

## Results

### General clinical characteristics of the recruited subjects

In the present study, 30 women from control group were 32–52 years old with a mean age of 43.60 ± 5.35. In the CIN group, age ranged from 26–46 years old with a mean age of 42.93 ± 5.35 years old; and in the CSCC group age ranged from 37–59 years old with a mean age of 40.67 ± 6.29 years old. No significant difference was found between these subject groups (P>0.05).

### Expression of YY1, E-cadherin and HPV16 E6 in cervical tissues

**Fig 1:**
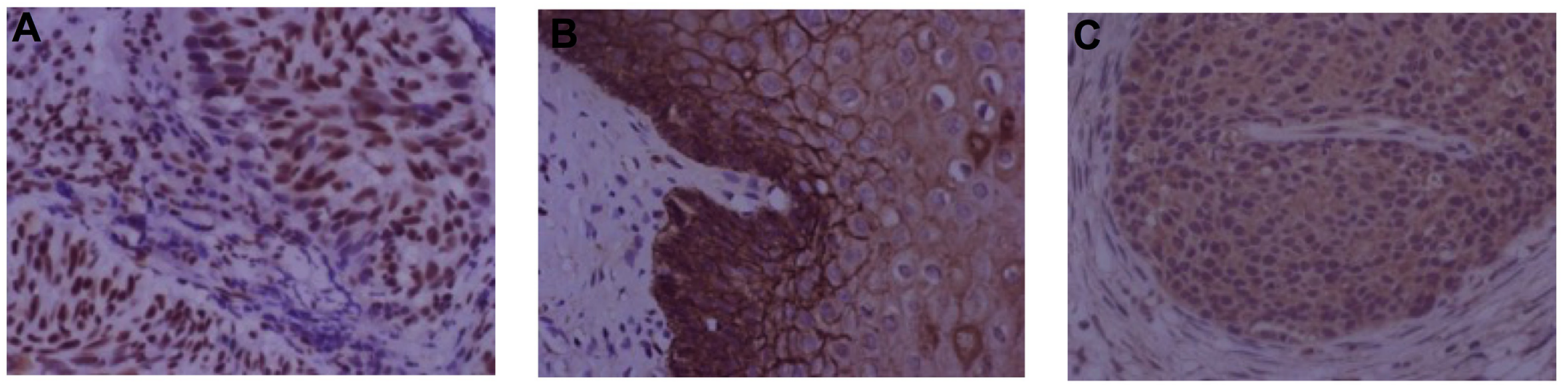
Protein expression patterns of YY1, E-cadherin and HPV16 E6 in cervical tissues. Representative images of immunohistochemistry (400x) for (A) YY1, (B) E-cadherin and (C) HPV16 E6 in normal cervical tissues.

The expression of YY1, E-cadherin and HPV16 E6 were detected by IHC. The negative control images with corresponding isotypes (YY1, E-cadherin and HPV16 E6) were shown in S1 Fig. YY1 expression was detected in all tissues examined, and the expression of YY1 was mainly located in the nucleus (Fig 1A). E-cadherin was also detected in all tissues examined, and its expression mainly located in the cell membrane (Fig 1B). For HPV16 E6 expression, the expression of HPV16 E6 was mainly found in the cytosol (Fig 1C), and the HPV16 E6 expression was detected in 81.1% (73/90) of all tissues examined, 66.67% (20/30) in the normal tissue group, 83.33% (25/30) in the CIN group, and 93.33% (28/30) in the CSCC group.

**Fig 2:**
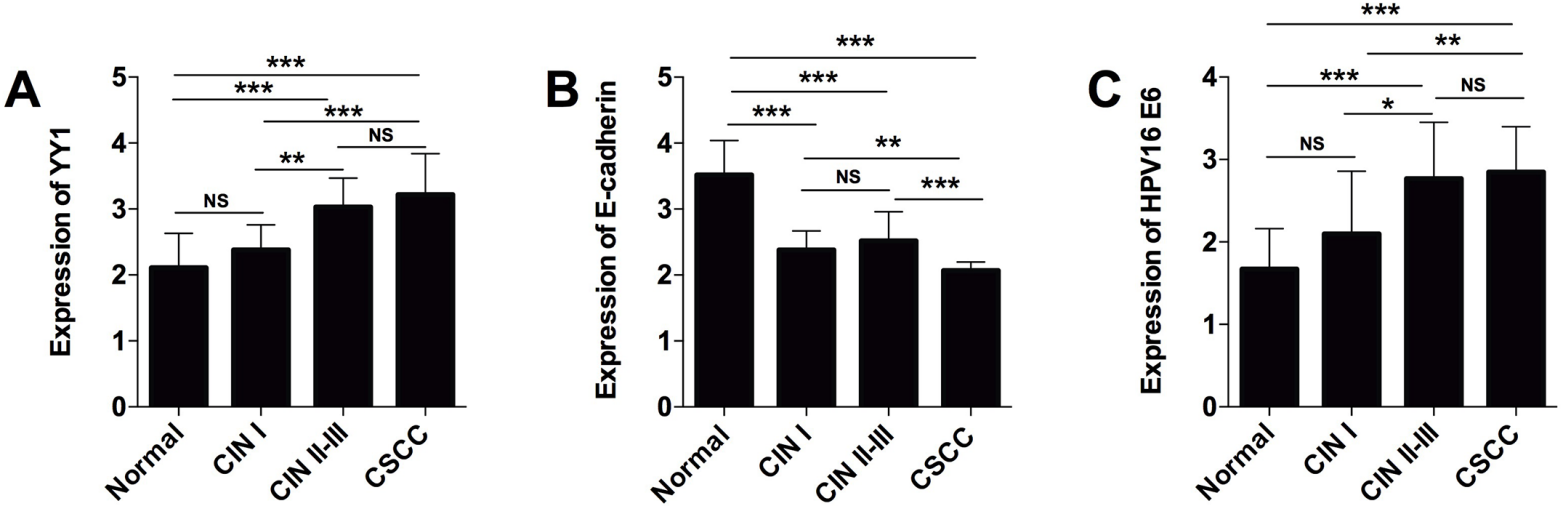
Summary of YY1, E-cadherin, HPV16 E6 H-score results in cervical tissues from different histological groups. H-scores for (A) YY1, (B) E-cadherin and (C) HPV16 E6 in normal group (n = 30), CIN I group (n = 15), CIN II-III group (n = 15) and CSCC group (n = 30). Significant differences between groups were shown as *P<0.05, **P<0.01 and ***P<0.001. NS = not significant.

**Fig 3:**
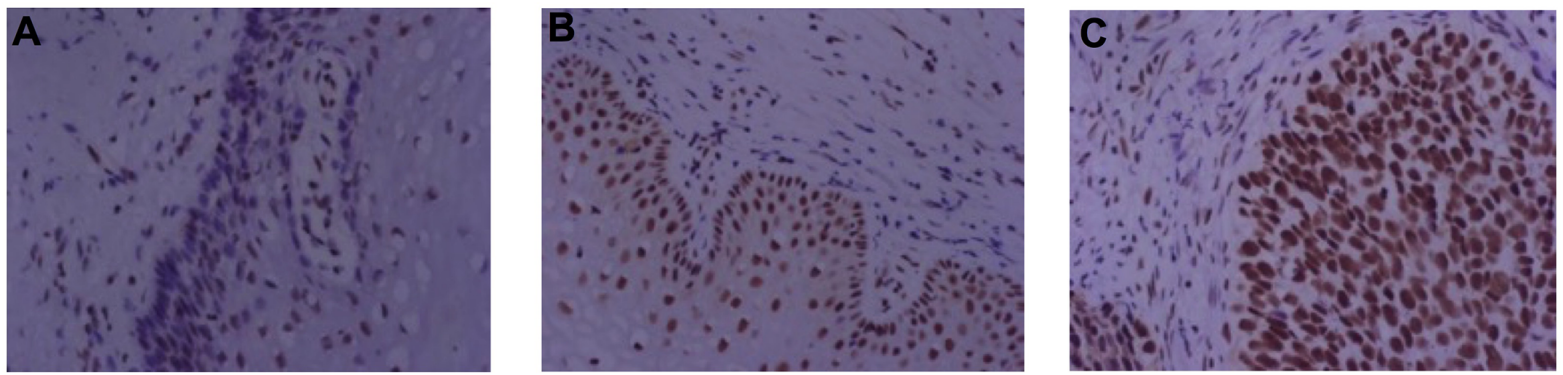
Protein expression patterns of YY1 in cervical tissues. Representative images of immunohistochemistry (400x) for YY1 in (A) normal cervical group, (B) CIN group, and (C) CSCC group.

The expression of YY1, E-cadherin, and HPV16 E6 H-scores were compared between the normal tissue group, CIN group and CSCC group. As shown in Fig 2A, there was no significant difference in YY1 expression between the normal group and the CIN I group (P>0.05), and the expression of YY1 in the CIN II-III group was significantly higher than that in normal group and CIN I group (P<0.05). The expression of YY1 in the CSCC group was also significantly higher than that in the normal group and the CIN I group (P<0.05), while no significant difference was detected in terms of YY1 expression between the CIN II-III group and the CSCC group (P>0.05). The representative IHC images of YY1 in normal tissue, CIN and CSCC groups are shown in Fig 3.

**Fig 4:**
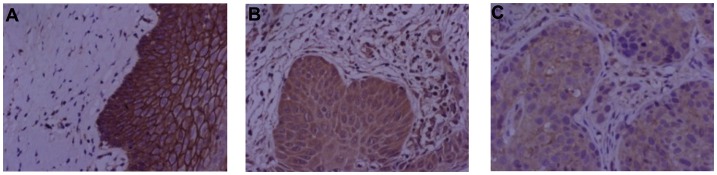
Protein expression patterns of E-cadherin in cervical tissues. Representative images of immunohistochemistry (400x) for E-cadherin in (A) normal group, (B) CIN group, and (C) CSCC group.

The expression results of E-cadherin are presented in Fig 2B, where there is significant difference of E-cadherin expression between CIN I and CIN II-III groups (P>0.05). The expression of E-cadherin in both CIN I and CIN II groups were significantly lower than that of the normal group (P<0.05). Additionally, the expression of E-cadherin in the CSCC group was significantly lower than that in normal, CIN I and CIN II-III groups (P<0.05). The representative IHC images of E-cadherin in normal, CIN, and CSCC groups were present in Fig 4.

**Fig 5:**
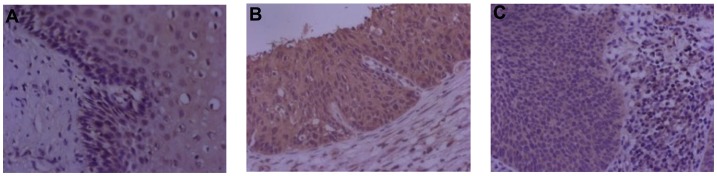
Protein expression patterns of HPV16 E6 in cervical tissues. Representative images of immunohistochemistry (400x) for HPV16 E6 in (A) normal group, (B) CIN group, and (C) CSCC group.

The expression of HPV16 E6 was also compared among different tissue groups. As shown in Fig 2C, the expression of HPV16 E6 in CIN II-III group was significantly higher than in normal group and CIN I group (P<0.05). While there was no significant difference detected in terms of HPV16 E6 expression between normal and CIN I groups (P>0.05). The expression of HPV16 E6 in CSCC group was also significantly higher than that in the normal group and CIN I group (P<0.05), while no significant difference for HPV16 E6 expression was found between CIN II-III group and CSCC group (P>0.05). Fig 5 presents representative IHC images of HPV16 E6 in control, CIN, and CSCC groups.

### Correlation between YY1 expression and, E-cadherin or HPV16 E6, expression in cervical tissues

**Fig 6:**
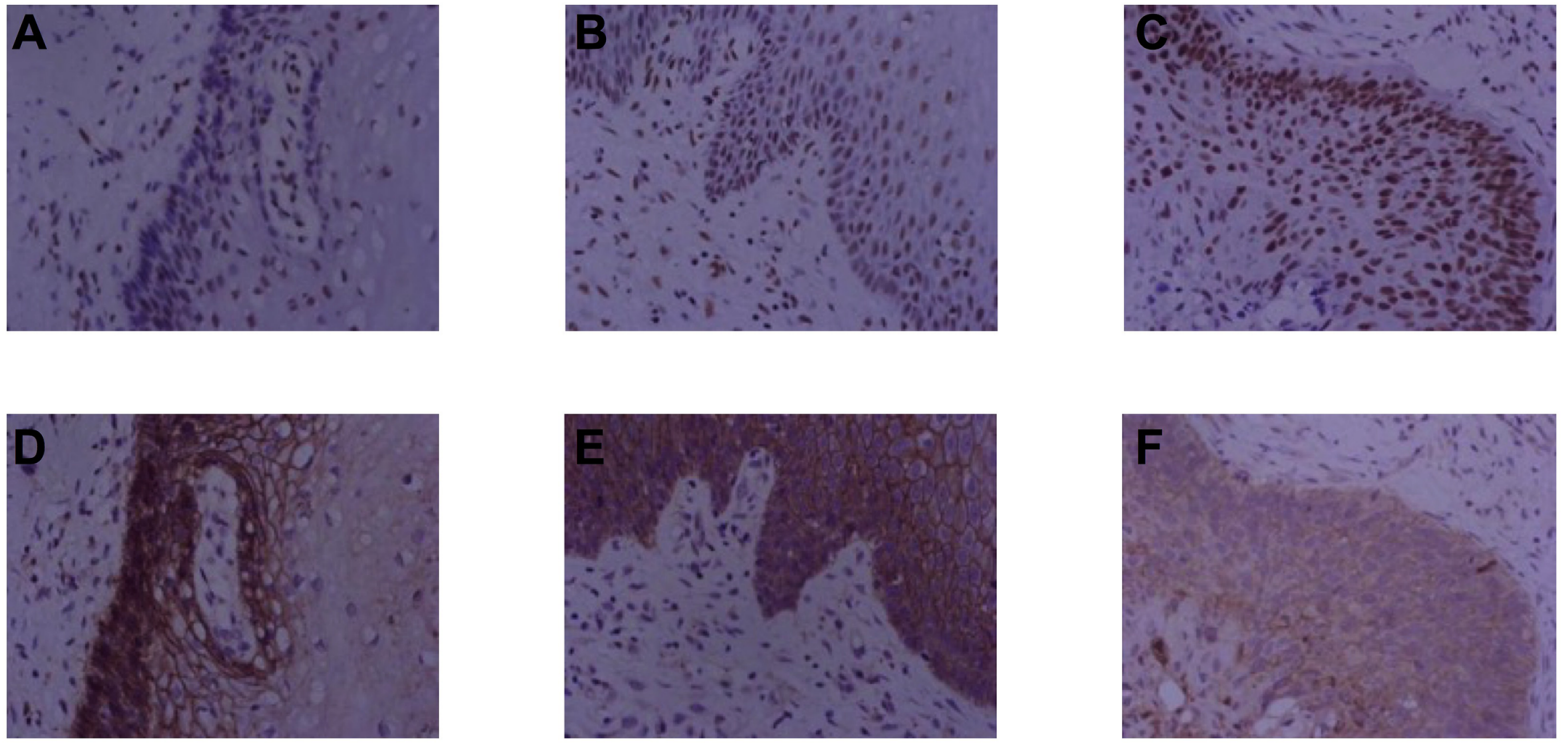
Protein expression patterns of YY1 and E-cadherin in cervical tissues. The representative images of sequential sections (400x) for YY1 in (A) normal group, (B) CIN group, and (C) CSCC group, and E-cadherin in (D) normal group, (E) CIN group, and (F) CSCC group.

The correlation between YY1 expression and, E-cadherin or HPV16 E6 expression in all cervical tissues was analyzed by Spearman's rank correlation test. As shown in Table 1, YY1 expression was negatively correlated with E-cadherin expression in all cervical tissues examined (r = -0.523, P<0.001). However, YY1 expression was positively correlated with HPV16 E6 expression in all cervical tissues examined (r = 0.444, P<0.001). Representative images of sequential sections for YY1 and E-cadherin staining in control, CIN, and CSCC groups is shown in Fig 6.

**Table 1:** The correlation between YY1 and E-cadherin/HPV16 E6 in the recruited patients.

Parameters	r values	P values
E-cadherin	-0.523	<0.001
HPV16 E6	0.444	<0.001

### Correlation between YY1 expression and clinical pathological factors in CSCC group

The CSCC group was divided into two groups based on tumor differentiation i.e. well or moderately differentiated vs. poorly differentiated. There was no significant difference in *YY1* expression between the two groups (Table 2, P = 0.7631). In addition, the CSCC group was also divided into two groups according to FIGO stage i.e. I_A_-I_B1_ vs. I_B2_-II_A_. The expression of YY1 in I_B2_-II_A_ group was significantly higher than that in I_A_-I_B1_ group (Table 2, P<0.001). Spearman's rank correlation analysis further revealed that the YY1 expression was not significantly correlated with tumor differentiation (r = 0.7744, P>0.05), while its expression was positively correlated with more advanced FIGO stage (r = 0.6771, P<0.001).

**Table 2:** The expression of YY1 in patients with cervical squamous cell carcinoma subgrouped by clinical pathologic factors.

Clinical pathologic factors	Classification	Patient number	H-score	P values
Tumor differentiation	Well or moderately differentiated	13	3.18 ± 0.63	0.7631
	Poorly differentiated	17	3.25 ± 0.62	
FIGO stage	I_A_, I_B1_	12	2.72 ± 0.38	<0.001
	I_B2_, II_A_	18	3.55 ± 0.50	

## Discussion

YY1 is a ubiquitously distributed transcriptional factor that belongs to the GLI-Kruppel class of zinc finger proteins, and it is located in the telomeres of chromosome 14q32.2. YY1 can act as either a transcriptional activator or transcriptional repressor in gene expression regulation [21, 22]. Mounting evidence from cancer studies has revealed that YY1 is involved cell cycle regulation, cell proliferation and apoptosis as well as modulation of oncogenes and tumor-suppressor genes expression [23]. YY1 was found to be involved in tumor invasion and migration by regulating the epithelial-mesenchymal transition (EMT) process, and YY1 promoted EMT in tumor cells via NF-κB/Snail/YY1/RKIP/PTEN signaling [24]. The abnormal expression of YY1 has been reported in various types of cancers, and its abnormal expression has been linked to poor prognosis in cancer patients [25]. Overexpression of YY1 predicted poor prognosis in breast cancer and non-Hodgkin lymphoma, while the YY1 overexpression was found to be inversely correlated with poor prognosis in prostate cancer [25]. Studies have also demonstrated up-regulation of YY1 in the cervical cancer [18], which implicates YY1 overexpression in poor prognosis prediction in cervical cancer.

In the present study, the expression of YY1, E-cadherin and HPV16 E6 in normal cervical tissues, CIN tissues and CSCC tissues at histological level using IHC for the first time. The correlation between YY1 and E-cadherin/HPV16 E6 expression was also explored. YY1 was expressed in the cell nucleus, in agreement with its functional role as a transcriptional regulator. We also found that YY1 was up-regulated in CSCC tissues when compared to normal cervical or CIN I tissue, suggesting a potential relationship between YY1 and the pathogenesis of cervical cancer. The expression of YY1 in CSCC tissues was positively correlated with advance FIGO stage, but was not correlated with the tumor differentiation, suggesting that YY1 may also be associated the CSCC infiltration.

E-cadherin belongs to the cadherin protein family and is mainly expressed in the membrane of the epithelial cells. It is critical for the formation and maintenance of adherent junctions in areas of epithelial cell-cell contact. E-cadherin is an important tumor suppressor and down-regulation of E-cadherin can promote EMT, a critical process for the metastasis and invasion for malignant tumors originating in the epithelial [14]. As an important regulator of EMT, loss of E-cadherin suppresses cell adhesion and polarity, which promotes EMT in tumor cells. Studies have suggested that E-cadherin may serve as an important biomarker for tumor malignancy, metastasis, recurrence and prognosis in cervical cancer [12].

In the present study, E-cadherin was found in the cell membrane of all the cervical tissues examined, meaning E-cadherin may be related to cell adhesion mediation. Our results demonstrated that E-cadherin expression in CSCC tissues was significantly lower than that in normal cervical tissues and CIN tissues. Additionally, down-regulation of E-cadherin may suppress cell adhesion and promote EMT, which can subsequently enhance tumor invasion and metastasis in CSCC. In our study, though no significant difference for E-cadherin expression between CIN I and CIN II-III groups was detected, the expression of E-cadherin in CIN II-III group tended to be higher than in the CIN I group. This finding is contrary to hypothesis that decreased E-cadherin expression is inversely correlated with increased malignancy in cervical cancer. Such a contradiction may be explained by the limited number of samples and variations in H-scores, thus, in future studies, more clinical samples should be examined to confirm the relationship between E-cadherin expression and progression of cervical cancer.

The expression of E-cadherin is regulated by various cellular factors in the tumor. The promoter region of human E-cadherin contains two E-box, and Snail competitively binds to E-box as a promoter, which in turn suppresses the expression of E-cadherin. In addition, MMP-9 has been found to suppress the expression of E-cadherin [26]. In breast cancer, YY1 suppresses the expression of E-cadherin via NF-κB/Snail/YY1/RKIP/PTEN signaling [24], as well as via the methylation process [27]. In this regard, we hypothesized that YY1 suppresses the expression of E-cadherin in cervical cancer. In the present study, we found that the expression of YY1 was negatively correlated with the expression of E-cadherin. These findings imply that YY1 promotes tumor metastasis in cervical cancer via suppression of E-cadherin expression. The underlying molecular mechanism involving the interaction between YY1 and E-cadherin may require further investigations.

HPVs are a group of circular dsDNA viruses that infect epithelia cells, and recipient cell entering the M phase is critical for the infection of HPV [28], and HPV-DNA integration into cellular chromatin is usually for pathogenesis in HPV-related cancer [29]. The HPV16 E6 protein is the major proto-oncogenic protein of this virus; it is involved in many critical pathogenesis pathways in cervical cancer. Studies have shown that HPV16 E6 can bind to E6 associated protein (E6AP) to form the E6-E6AP complex; the E6-E6AP complex subsequently binds to p53, causing it to degrade [30]; HPV 16 E6 expression inhibited the stabilization of p53 and promoted tumorigenesis via genome destabilization [31, 32]. In addition, HPV 16 E6 protein can bind Bak to stimulate Bak degradation and reduce Bak-induced apoptosis, which may contribute to the oncogenic potential of the virus [33]. Studies from Klingelhutz et al., also showed that HPV16 E6 protein can induce telomerase activation [34]. Evidence from animal studies showed that HPV E6 and E7 proteins acted synergistically to cause head and neck cancer in mice [35]. Additionally, studies have reported that the E-cadherin promoter is repressed in cells expressing HPV16 E6, resulting in fewer E-cadherin transcripts. E-cadherin regulation by HPV16 E6 has been suggested to contribute to viral immune evasion, as adhesion between keratinocytes and the epidermal antigen presenting cells are E-cadherin-mediated [36]. Additional studies have also shown that HPV16 E6 regulates the expression of E-cadherin via targeting Wnt/β-catenin signaling pathway [37]. HPV16 E6 was shown to activate human telomerase reverse transcriptase, which was suggested as an important mechanism of HPV16 E6-mediated tumorigenesis [38].

Our results demonstrated that the expression of HPV16 E6 in CSCC tissues was higher than that in normal cervical and CIN I tissues, suggesting that HPV16 E6 may be involved in the CSCC pathogenesis. In addition, the expression of HPV16 E6 in CIN II-III tissues was higher than that in CIN I tissues, but was similar to those levels found in CSCC tissues, suggesting that patients with CIN II-III may have high-risk of progressing into CSCC. Correlation analysis further revealed that the expression of YY1 in CSCC tissues was positively correlated with expression of HPV16 E6, suggesting that YY1 can promote the expression of HPV16 E6 in cervical cancer pathogenesis. In *in vitro* study, YY1 was found to repress HPV16 E6 promoter by quenching the AP-1 activity in HeLa cells [39]. However, the E6 promoter of extrachromosomal HPV16 DNA in cervical cancer escaped cellular repression by mutating YY1 target sequences [40]. Further experimentation suggested that mutation of YY1-motifs long control regions is one of the mechanisms for enhancement of HPV oncogene expression during cancer cell progression [41]. More mechanistic studies should be performed to clarify this discrepancy about the interaction between YY1 and HPV16 E6.

In summary, the present study demonstrated that the abnormal expression of YY1, E-cadherin and HPV16 E6 were associated with cervical squamous cell carcinoma progression. There was a negative correlation between YY1 expression and E-cadherin expression, and positive correlation between YY1 expression and HVP16 E6 expression in cervical cancer tissues. These results suggest that YY1 may play an important role in the development of cervical cancer by modulating the expression of E-cadherin and HPV16 E6.

## Supporting information

S1 Fig Negative control staining images for YY1, E-cadherin and HPV16 E6 in cervical tissues. Representative images (400x) of isotype controls for YY1 staining in (A) normal cervical group, (B) CIN group, and (C) CSCC group; representative images (400x) of isotype controls for E-cadherin staining in (D) normal cervical group, (E) CIN group, and (F) CSCC group; representative images (400x) of isotype controls for HPV16 E6 staining in in (G) normal cervical group, (H) CIN group, and (I) CSCC group.
(DOCX)

S2 Fig Ethics approval Page 1. (TIF)

S3 Fig Ethics approval Page 2. (TIF)

S4 Fig Ethics approval Page 3. (TIF)
