# Supplementary material for: Expression of Yin Yang 1 in cervical cancer and its correlation with E-cadherin expression and HPV16 E6
Source: PLoS One. 2018 Feb 22;13(2):e0193340. doi: 10.1371/journal.pone.0193340 (PMC5823405; doi:10.1371/journal.pone.0193340)
Supplement: S1 Fig — Representative images (400x) of isotype controls for YY1 staining in (A) normal cervical group, (B) CIN group, and (C) CSCC group; representative images (400x) of isotype controls for E-cadherin staining in (D) normal cervical group, (E) CIN group, and (F) CSCC group; representative images (400x) of isotype controls for HPV16 E6 staining in in (G) normal cervical group, (H) CIN group, and (I) CSCC group. (DOCX) [file pone.0193340.s001.docx]

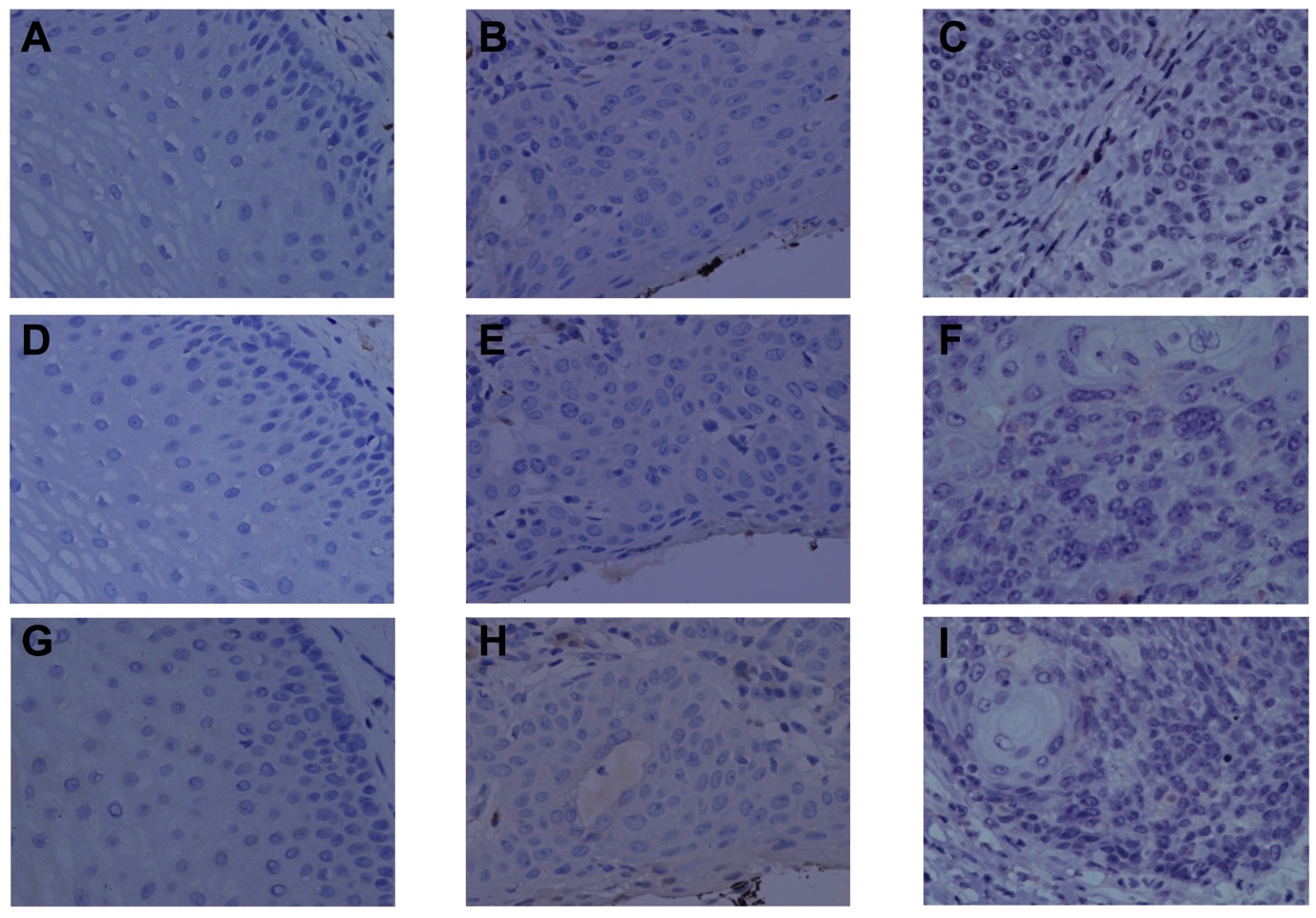


**Figure S1. Negative control staining images for YY1, E-cadherin and HPV16 E6 in cervical tissues.** Representative images (400x) of isotype controls for YY1 staining in (A) normal cervical group, (B) CIN group, and (C) CSCC group; representative images (400x) of isotype controls for E-cadherin staining in (D) normal cervical group, (E) CIN group, and (F) CSCC group; representative images (400x) of isotype controls for HPV16 E6 staining in in (G) normal cervical group, (H) CIN group, and (I) CSCC group.
