# Supplementary figures and images for: Expression of Yin Yang 1 in cervical cancer and its correlation with E-cadherin expression and HPV16 E6
Source: PLoS One. 2018 Feb 22;13(2):e0193340. doi: 10.1371/journal.pone.0193340 (PMC5823405; doi:10.1371/journal.pone.0193340)

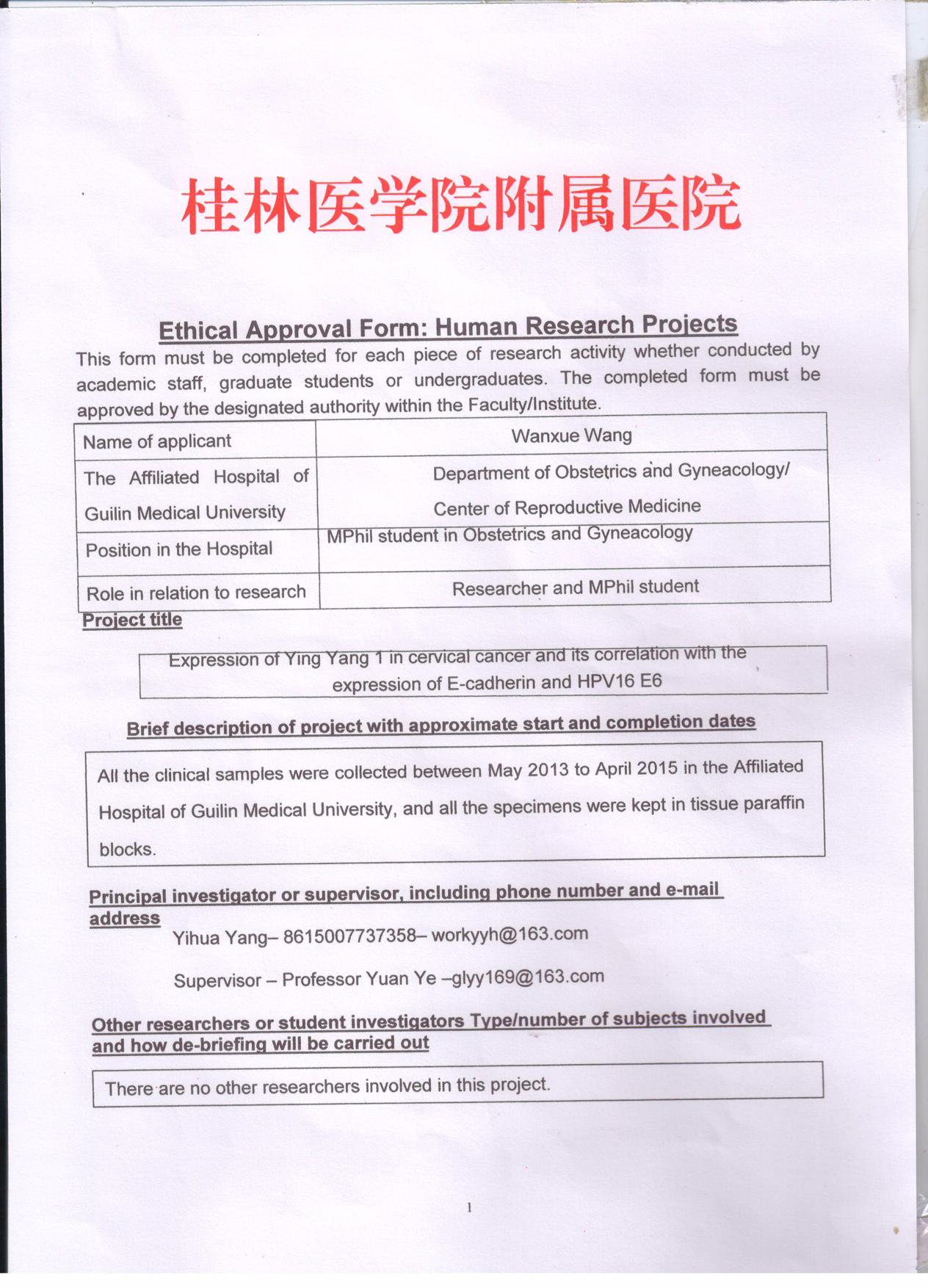

Supplement: S2 Fig — (TIF) [file pone.0193340.s002.tif]

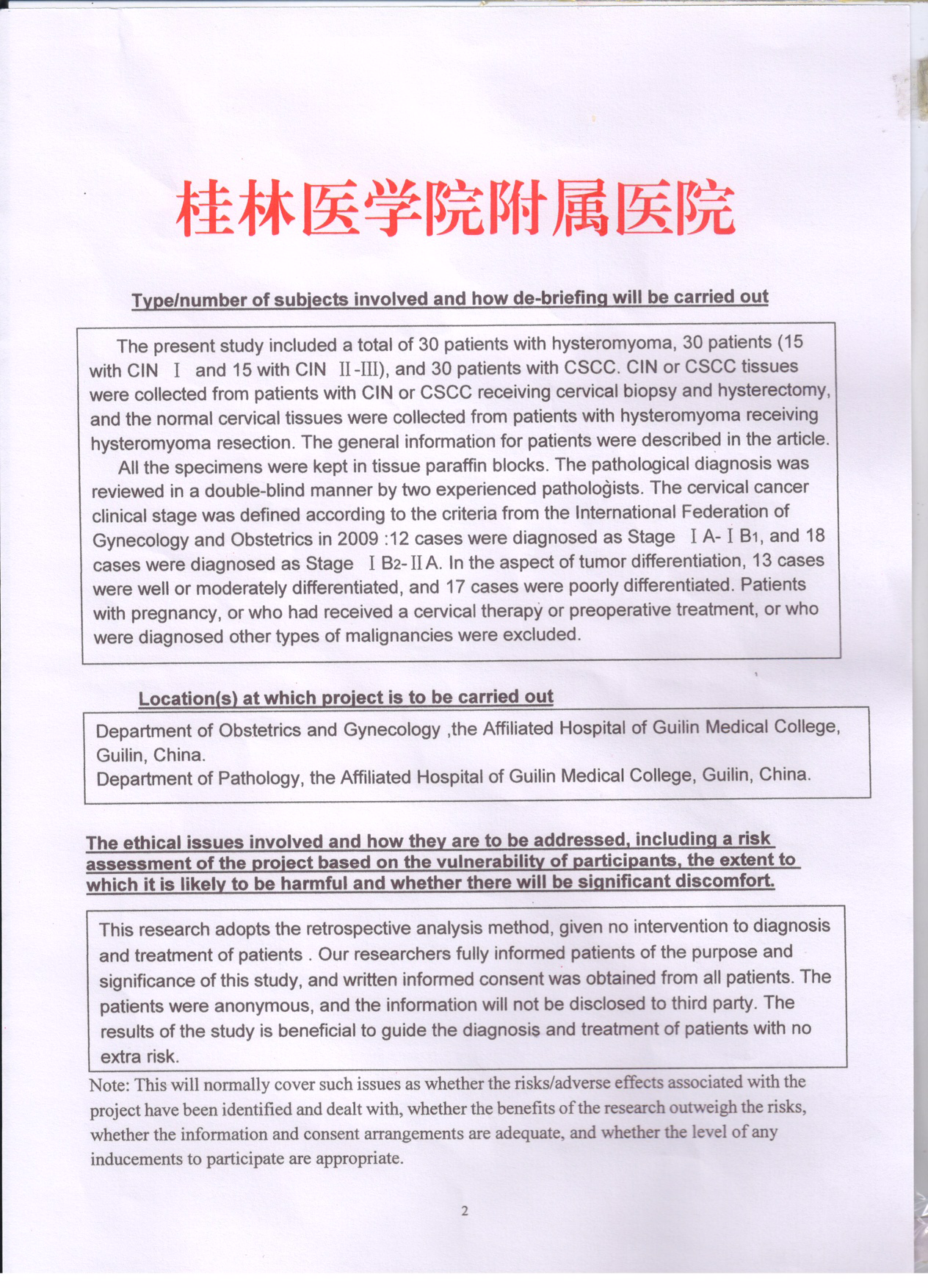

Supplement: S3 Fig — (TIF) [file pone.0193340.s003.tif]

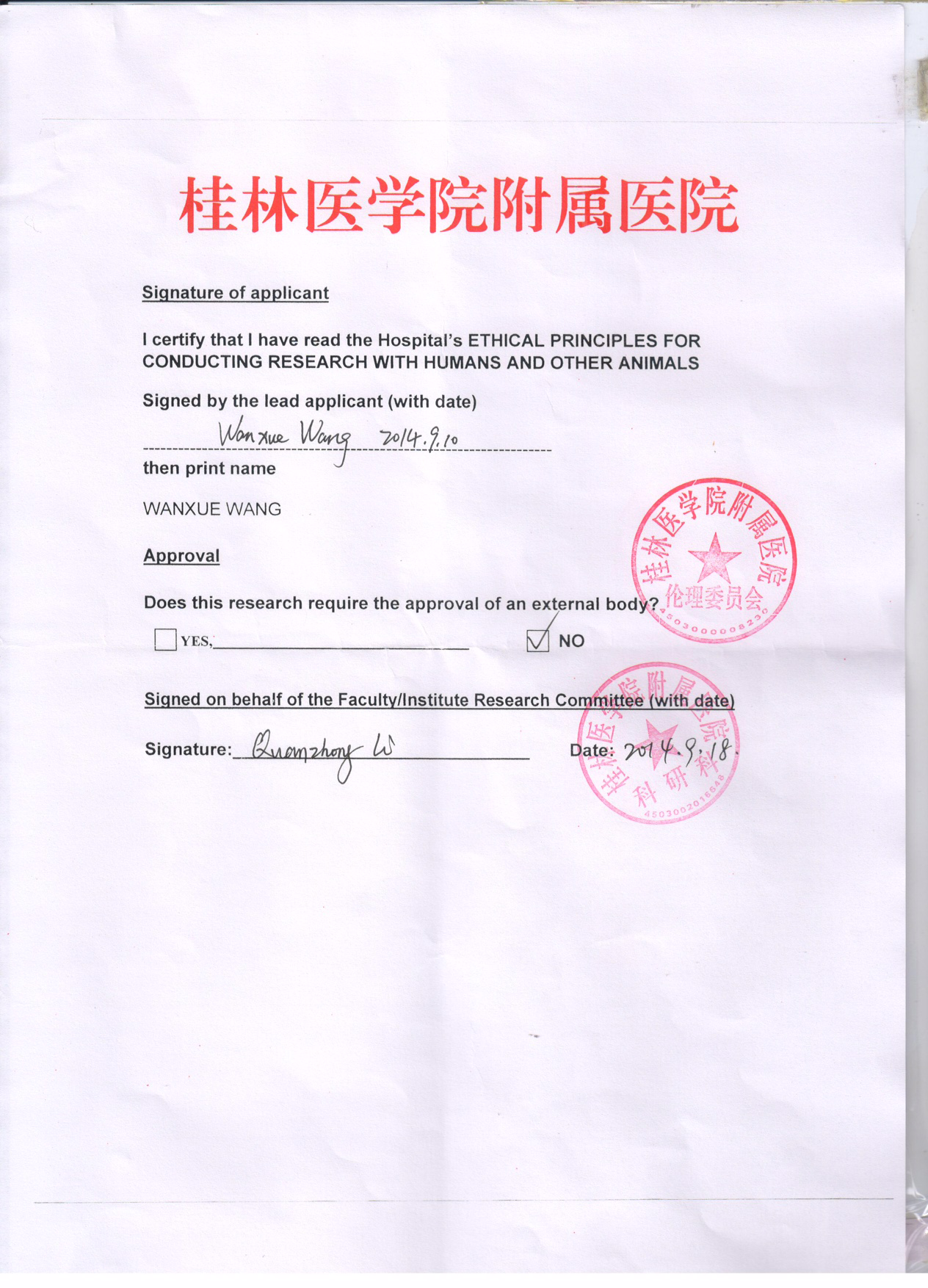

Supplement: S4 Fig — (TIF) [file pone.0193340.s004.tif]
